# Systematic review of PSA reference intervals in the gender diverse population with prostates

**DOI:** 10.1111/bju.16825

**Published:** 2025-06-22

**Authors:** Rose Hall, Rue Ball, Elizabeth Bancroft, Rosalind Eeles, Alison May Berner

**Affiliations:** ^1^ The Institute of Cancer Research London UK; ^2^ The Royal Marsden NHS Trust London UK; ^3^ Queen Mary University of London London UK; ^4^ St Thomas’ Hospital London UK

**Keywords:** diagnosis, gender‐affirming hormone therapy, non‐binary, prostate cancer, prostate‐specific antigen, screening, transgender

## Abstract

**Objectives:**

To determine mean/median serum total prostate‐specific antigen (PSA) levels in transgender women and non‐binary people with prostates (TWNBPP) who have received gender‐affirming hormone therapy (GAHT) or an orchidectomy. The secondary objective was to identify other quantitative information that influences PSA levels in this population.

**Methods:**

Systematic review of existing publications from primary studies published in English, excluding case reports and guidelines. Included studies: TWNBPP who have received GAHT/post‐orchidectomy, without a diagnosis of prostate pathology, with recorded serum PSA levels. MEDLINE and Embase databases were searched, up to July 2024.

**Results:**

Four papers met the inclusion criteria, with 290 participants. Two papers measured the mean PSA level after 4 and 12 months of GAHT (mean [range] age 30 [18–45] years). A third paper measured the mean PSA level after a median of 9 years of GAHT (mean [range] age 40.1 [19–67] years). The fourth study measured 852 PSA levels in 210 participants receiving oestradiol therapy, over a 23‐year period (mean [range] age 60 [40–79] years). The mean and median PSA levels ranged from 0.020 to 0.525 ng/mL. Meta‐analysis of these data was unfeasible, due to low quantity, comparability, and quality of the studies.

**Conclusions:**

Existing data for serum PSA reference intervals for TWNBPP without prostate pathology were from four studies and cannot be used to make clinical recommendations. The evidence indicates that GAHT in TWNBPP lowers PSA levels from baseline, below expected levels for age‐matched cisgender controls. Not all TWNBPP over the age of 40 years should be offered PSA testing; however, those with a genetic predisposition, family history, or symptoms of prostate cancer, may request or be offered a PSA test. There are currently no clinical PSA thresholds to guide interpretation of PSA levels in TWNBPP when being evaluated for suspected prostate cancer or for those seeking PSA testing.

**Abbreviations**
AXIS: Appraisal of Cross‐Sectional Studies (tool)
cis: cisgender
GAHT: gender‐affirming hormone therapy
GAS: gender‐affirming surgery
GRADE: Grading of Recommendations Assessment, Development, and Evaluation (tool)
IQR: interquartile range
SE: standard error
trans: transgender
TWNBPP: trans women and non‐binary people with a prostate

## Introduction

Prostate cancer is the second most common cancer in cisgender (cis) men worldwide [1], but all people who were assigned male at birth have a prostate and are thus susceptible. PSA is a protein produced by the prostate gland and more of it may be produced in benign and malignant conditions of the prostate.

According to IPSOS Mori 2023, 1% of the world population identify as transgender (trans), with a gender identity not consistent with the sex they were assigned at birth [2, 3]. This number is increasing, due to increased disclosure facilitated by societal awareness and acceptance, and access to support for transition [2, 4, 5]. Trans women and non‐binary people with a prostate (TWNBPP) include trans women (those having a female gender identity but who were assigned male at birth) and non‐binary people (those who do not identify exclusively as a male or female) [3]. Trans people may have gender‐affirming therapy to change their sex characteristics [3], which include gender‐affirming hormone therapy (GAHT) (Table 1 [6]) and gender‐affirming surgery (GAS) [3]. GAHT for TWNBPP consists of oestradiol, with or without antiandrogens, and individuals may undergo bilateral orchidectomy as part of GAS. The prostate is always retained after GAS [6].

**Table 1:** Mode of delivery and dose of GAHT drugs used in TWNBPP.

Class, route and drug	Standard dose
Oestrogen	
Oral	
Oestradiol valerate	2.0–6.0 mg daily
Transdermal	
Oestradiol transdermal patch (new patch placed every 3–4 days)	0.025–0.2 mg daily
Parenteral	
Oestradiol valerate or cypionate	5–30 mg IM every 2 weeks
	2–10 mg IM every week
Antiandrogens	
Oral	
Spironolactone	100–300 mg daily
Cyproterone acetate*	10 mg daily*
Parenteral	
GnRH agonist	3.75 mg SC monthly
	11.25 mg SC 3‐monthly

IM, intramuscular; SC, subcutaneous.

Adapted from Hembree et al. [6]

* Note that while doses of 25–50 mg/day of cyproterone acetate were recommended in the 2017 Endocrine Society Guidelines, subsequent evidence suggests that doses of 25 mg/day or more long‐term is associated with increased risk for meningioma. Studies have shown that doses of 10 mg/day achieve sufficient antiandrogenic effects, with a lower risk of side effects [43].

According to National Institute for Health and Care Excellence (NICE) guidelines, cis men should be promptly referred to a specialist if their PSA concentration is above an age‐dependant cut‐off that ranges from 2.5 to 6.5 ng/mL across ages 50–79 years [7]. Most primary care physicians use a single age‐dependent PSA cut‐off for further investigation (e.g., a 60‐year‐old cis man with a PSA of ≥4 ng/mL may be referred for MRI) [7]. These cut‐offs are based on the age‐dependent upper reference limit for PSA observed in healthy cis men. However, the PSA test may be within reference range in the presence of prostate cancer, or PSA levels may be high for reasons other than cancer, and it does not distinguish between indolent or advanced prostate cancer. The Prostate Cancer Prevention Trial included 18 882 cis men, and 15% of those with a PSA <4 ng/mL (the reference range used), were found on biopsy to have prostate cancer, of which 15% had advanced disease (Gleason Score >7) [8]. Therefore, guidelines set a PSA cut‐off for further investigations that include a high number of, but not all, prostate cancer cases; to reduce patient harm from overdiagnosis and overtreatment. The ideal PSA cut‐off would capture high‐risk and advanced disease, avoiding investigation of indolent or low‐risk disease.

Gender‐affirming hormone therapy and orchidectomy reduce serum testosterone levels due to suppression of endogenous testosterone, which consequently reduces serum PSA [9, 10]. This also means that incidence of prostate cancer in TWNBPP who have received GAHT or orchidectomy are two to five times lower than cis men, with an average age of diagnosis of 61 and 64 years [11, 12]. TWNBPP who develop clinically significant prostate cancer (life‐limiting disease) may or may not present with a PSA concentration above the cut‐off for cis men at diagnosis [9, 11, 13, 25].

Using cis male PSA reference intervals in TWNBPP sets a higher PSA threshold for referral for further investigations. Given the lower incidence, this may be appropriate. However, prostate cancer cells utilise testosterone to drive growth and proliferation, until a certain point known as ‘castration resistance’, where the tumour cells evolve to be able to grow in the absence of testosterone [14]. Through occurring in the absence of testosterone, prostate cancer is castrate resistant when diagnosed in TWNBPP who are receiving GAHT. In the treatment of prostate cancer, castrate‐resistant disease confers a poorer prognosis, due to fewer treatment options [15].

There is conflicting evidence about whether prostate cancer in TWNBPP is more likely to be aggressive or is comparable to age‐matched cis men. Studies show TWNBPP who are diagnosed with prostate cancer likely commenced GAHT at a later age, with the possibility of pre‐existing prostate cancer [11]. Therefore, it could be argued that TWNBPP starting GAHT over the age of 40 years should have a PSA test as a baseline, pre‐GAHT initiation. TWNBPP who started GAHT at a younger age are less likely to develop prostate cancer than those who started GAHT later in life, due to individuals over the age of 40 years having a greater risk of pre‐existing prostate cancer [11, 12]. Nik‐Ahd et al. [9] and Manfredi et al. [16] describe TWNBPP having higher mortality and more aggressive disease in those receiving oestrogen [12]. They suggest theories for causation are disease that has evolved in a low‐testosterone setting, the presence of alpha oestrogen receptors on the prostate (stimulates prostate carcinogenesis, as opposed to beta oestrogen receptors, which are anti‐neoplastic) [17], or barriers to care and investigations causing late diagnosis. However, these studies have a high risk of bias given the small sample size, poor sampling methods, and literature review study design [18, 19]. Meagher et al. [20] performed a retrospective cohort study with age‐ and PSA‐matched cis men and 199 trans women with prostate cancer, where the results showed that in 5 years of follow‐up after diagnosis, disease outcomes and mortality were comparable between both groups.

This review summarises existing evidence describing PSA reference ranges for TWNBPP receiving GAHT, with the aim of providing guidance for clinicians for when to refer symptomatic TWNBPP for further prostate cancer investigations and for screening advice for TWNBPP who seek screening due to increased prostate cancer risk.

## Objectives

The primary objective was to locate mean/median serum total PSA levels in TWNBPP who have received GAHT or an orchidectomy. The secondary objective was to identify how PSA levels were affected by other factors such as age at prostate cancer diagnosis, age at starting GAHT, how long received GAHT prior to diagnosis, type of GAHT regimen, and orchidectomy status.

## Methods

### Literature Review

A systematic literature search was conducted using the Preferred Reporting Items for Systematic Review and Meta‐Analyses (PRIMSA) guidelines [21]. The protocol was registered with the International Prospective Register of Systematic Reviews (PROSPERO) registration number CRD42024546609.

Study inclusion criteria were as follows:

- Study design: any primary study, including randomised controlled trials, case–control studies, cohort studies, or cross‐sectional studies
- Population: TWNBPP who must have received either GAHT or orchidectomy, and must not have been diagnosed with prostate cancer or other prostate pathology
- Observation: any study that included measurement of serum PSA concentration, and reported summary statistics for the PSA, these could include a reference interval, mean, median or SD
- Language: published only in English
- Publication date: any date prior to July 2024

We excluded secondary research publications such as reviews or guidelines, and we excluded case reports. Studies of TWNBPP with a diagnosis of prostate pathology were also excluded.

We searched for publications in the MEDLINE and Embase databases using the following keywords and subject headings: ‘Transgender’, ‘Transsexual’, ‘Transsexualism’, ‘Gender dysphoria’, ‘Gender identity disorder’, ‘Gender diverse’ and ‘Prostate specific antigen’ or ‘PSA’. Appendix S1 shows the full queries for both databases. We did not include ‘prostate cancer’ in our search‐terms because we only wanted PSA levels in TWNBPP without a diagnosis of prostate cancer, as a prostate cancer screening method. The databases were last searched on 16 July 2024. The references of included studies were screened for eligible publications. Two reviewers (R.H., R.B.) independently screened article titles and abstracts, then retrieved and reviewed the full text of apparently eligible publications. Included articles were agreed upon by both reviewers, any disagreements were arbitrated by a third reviewer (A.M.B.).

### Data Synthesis and Analysis

Two reviewers independently read the full text of the studies included and one reviewer (R.H.) extracted relevant data. Mean and median serum PSA levels (continuous variables) were compiled into Table 2 [22, 23, 24, 25], along with other relevant data. Meta‐analyses were unfeasible due to the low quantity of data, high heterogeneity between studies, incomparable study designs, and low statistical weighting of mean PSA levels without SD ranges.

**Table 2:** Table of papers and extracted data.

Papers and GAHT regimen	Age, years, mean (range)	Duration of GAHT, years	Number of participants	PSA levels – variable length of GAHT		
				Before initiating GAHT	4 months of GAHT	12 months of GAHT
Nik‐Ahd et al., 2024 [25] All participants received oestrogen therapy. Of these, 49% have received orchidectomy, 89% spironolactone, 39% progesterone, 30% finasteride, 3% goserelin, 5% leuprolide	60 (40–79)	Median (range) 4.7 (0.5–29.9)	*N* = 210 Multiple PSA values per participant, 852 PSA values	No mean PSA level. Median (IQR) all PSA levels (852 values) = 0.02 (0–0.2) ng/mL Median (IQR) first PSA level in each participant (210 values) = 0.08 (0–0.3) ng/mL 95th percentile PSA level = 0.6 ng/mL. Highest PSA level = 2.1 ng/mL. 36% of PSA levels were 0 or undetectable		
Slagter et al., 2006 [24] Group 1 (*N* = 20) ‐ oral ethinyl oestradiol + cyproterone acetate. Group 2 (*N* = 15) ‐ transdermal 17β‐oestradiol* + cyproterone acetate	30 (19–45)	Intervals 0, 4 and 12 months	*N* = 35	Mean (SE) 0.525 (0.044) ng/mL Median (range) 0.437 (0.156–1.174) ng/mL	Group 1 ‐ mean (SE) 0.032 (0.006) ng/mL Median (range) 0.026 (0–0.121) ng/mL Group 2 ‐mean (SE) 0.177 (0.072) ng/mL Median (range) 0.063 (0.011–0.995) ng/mL	Reports no significant difference between PSA levels at 4 months and 12 months of GAHT (data not included in paper)
Obiezu et al., 2000 [23] Group 1, 100 mg cyproterone acetate OD for 12 months. Group 2, transdermal 17β‐oestradiol* + 100 mg cyproterone acetate OD for 12 months. Group 3, oral oestradiol OD + 100 mg cyproterone acetate OD for 12 months	Group 1 – 34 (19–50) Group 2 – 31 (20–44) Group 3 – 30 (18–43)	Intervals 0, 4 and 12 months	Total *N* = 56 Group 1, *N* = 10 Group 2, *N* = 15 Group 3, *N* = 31	Group 1 ‐ median 0.333 ng/mL Group 2 ‐ median 0.272 ng/mL Group 3 ‐ mean 0.327 ng/mL (no SE)	Group 1 ‐ median 0.035 ng/mL Group 2 ‐ median 0.041 ng/mL Group 3 ‐ mean 0.058 ng/mL	Group 3 ‐ mean 0.015 ng/mL, median 0.010 ng/mL No values for Group 1 or 2 were reported
Jin et al., 1996 [22] All participants received oestrogen therapy. 12 received antiandrogens, 10 received progestin, four had orchidectomy	40.1 (19–67)	Median (range) 9 (0.25–16) years	*N* = 14	Mean serum PSA level, received GAHT for a median (range)of 9 (0.25–16) years Compared to the mean of 42 age‐matched healthy cis male controls Mean (SE) PSA level 0.1 (0.02) ng/mL compared to controls 1.1 (0.1) ng/mL		

OD, once daily.

* Administered twice weekly, receiving on average 100 μg/day.

Heterogeneity was assessed narratively, and studies were too few and too heterogenous to be compiled into a funnel plot to assess publication bias. Authors within two of the studies assessed correlation between PSA levels before and after GAHT treatment, using Spearman correlation coefficient.

### Risk of Bias

Two authors independently assessed the risk of bias in each of the studies using the Appraisal of Cross‐Sectional Studies (AXIS) tool for quality assessment of primary studies. Each study was classified into low, moderate, or high risk of bias and if there was a disagreement between authors R.H. and R.B. regarding classification between low and moderate risk of bias, the third reviewer (A.M.B.) arbitrated. The results of this are tabulated (Table 3 [22, 23, 24, 25]).

**Table 3:** The AXIS assessment of study quality.

Study:	1	2	3	4	5	6	7	8	9	10	11	12	13	14	15	16	17	18	19	20	Score, *n/N* (%)	Quality
Jin et al., 1996 [22]	Y	Y	N	Y	N	N	X	Y	X	Y	Y	Y	N	N	Y	Y	Y	N	X	Y	11/20 (55)	Low
Obiezu et al., 2000 [23]	Y	Y	N	X	X	X	X	Y	Y	Y	N	Y	N	N	Y	N	Y	N	X	X	8/20 (40)	Low
Slagter et al., 2006 [24]	Y	Y	N	Y	X	N	X	Y	Y	Y	Y	Y	N	N	Y	N	Y	N	X	Y	11/20 (55)	Low
Nik‐Ahd et al., 2024 [25]	Y	Y	Y	Y	Y	Y	X	Y	Y	Y	X	Y	N	N	Y	N	Y	Y	N	Y	16/20 (80)	High

N, No; X, Does not say Y, Yes. Key: Criteria: 1 – Were the aims of the study clear?, 2 – Was the study design appropriate for the stated aim?, 3 – Was the sample size justified?, 4 – Was the target/reference population clearly defined?, 5 – Was the sample frame taken from an appropriate population base so that it closely represented the target/reference population under investigation?, 6 – Was the selection process likely to select subjects/participants that were representative of the target/reference population under investigation?, 7 – Were measures undertaken to address and categorise non‐responders?, 8 – Were the risk factor and outcome variables measured appropriate to the aims of the study?, 9 – Were the risk factor and outcome variables measured correctly using instruments/measurements that had been trialled, piloted or published previously?, 10 – Is it clear what was used to determined statistical significance and/or precision estimates?, 11 – Were the methods (including statistical methods) sufficiently described to enable them to be repeated?, 12 – Were the basic data adequately described?, 13 – Does the response rate raise concerns about non‐response bias?, 14 – If appropriate, was information about non‐responders described?, 15 – Were the results internally consistent?, 16 – Were the results presented for all the analyses described in the methods?, 17 – Were the authors’ discussions and conclusions justified by the results?, 18 – Were the limitations of the study discussed?, 19 – Were there any funding sources or conflicts of interest that may affect the authors’ interpretation of the results?, 20 – Was ethical approval or consent of participants attained? Total is how many ‘Yes’ per study (‘Yes’ being favourable except for questions 13 and 19 where a ‘No’ is favourable and therefore treated as a ‘Yes’ in the total score). Criteria answered with ‘X’ are included as a ‘No’ in the total.

### Certainty of Evidence

Overall certainty of evidence from the studies was independently assessed by two reviewers using the Grading of Recommendations Assessment, Development, and Evaluation (GRADE) tool. There were no disagreements between reviewers and evidence certainty was classified as very low (Table 4).

**Table 4:** The GRADE assessment for the certainty of evidence from studies.

No. of participants and studies	Risk of bias	Imprecision	Inconsistency	Indirectness	Publication bias	Overall certainty of evidence
*N* = 290 Studies = 4	High	High	High	Moderate	Low	Very low

## Results

**Fig. 1:**
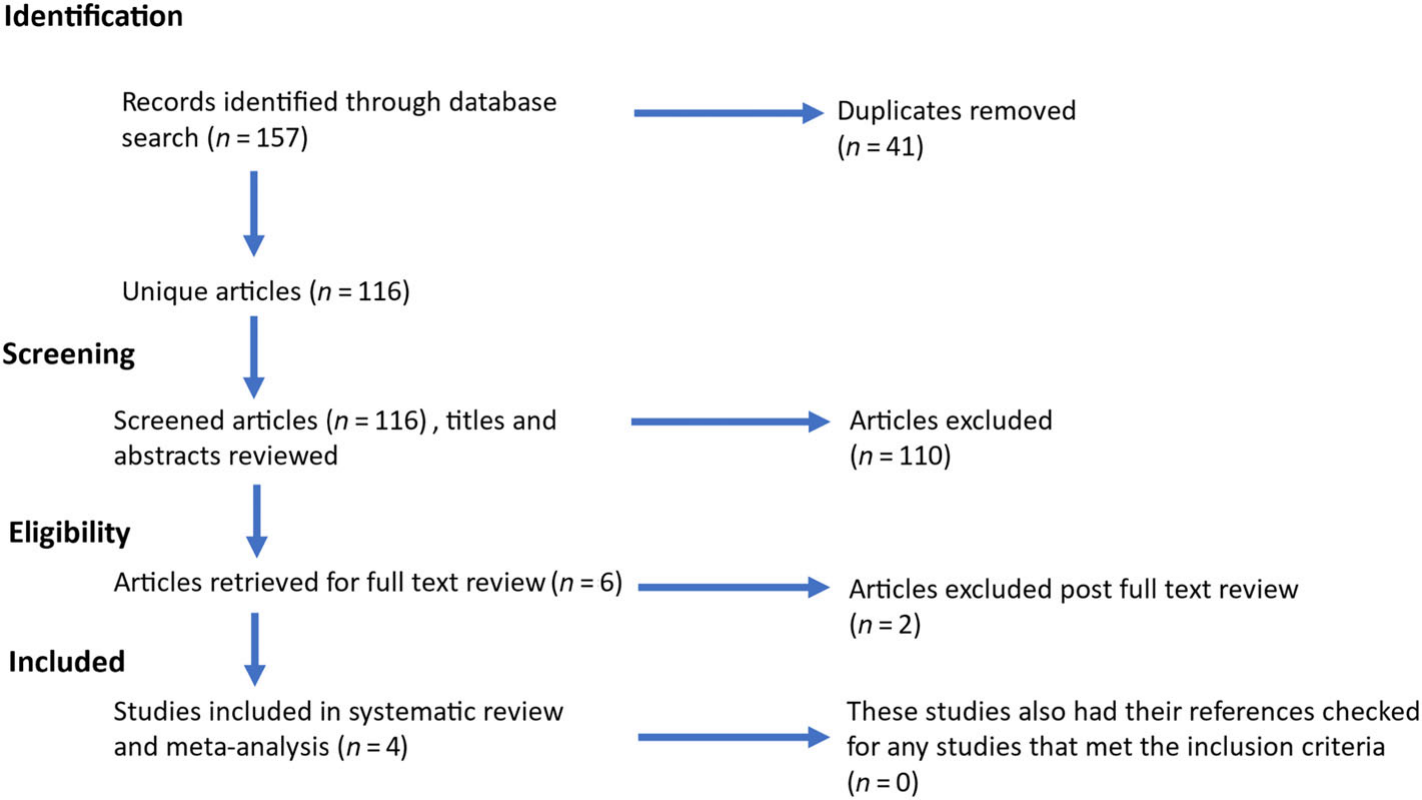
Search strategy.

The search queries returned 157 articles, of which 116 were unique articles. Of these, 110 articles were excluded due to not meeting the inclusion criteria, resulting in six articles. Full‐text review excluded a further two articles (Fig. 1), and four publications were identified that met the inclusion criteria (Table 2). These four studies were published between 1996 and 2024, with a combined total of 290 trans women participants [22, 23, 24, 25]. The locations and the years that the studies took place were not included in two of the four papers.

### Study Designs and Outcomes

Three studies published a mean serum PSA level, obtained from the participants within each study group. The fourth study published a median serum PSA with interquartile range (IQR). Groups were defined by the type of GAHT they received, none of the prospective studies stated if group allocation was participant choice or randomised [23, 24].

Jin et al. [22] conducted a cross‐sectional study that measured serum PSA levels in trans women receiving GAHT. There were 14 participants, with a mean (range) age of 40.1 (19–67) years, all of whom received GAHT for a median (range) of 9 (0.25–16) years. The authors compared the mean serum PSA in these participants to 42 age‐matched eugonadal healthy cis male controls. All 14 trans women were taking oestradiol, and 12 of them were also taking antiandrogen. Four had undergone orchidectomy. The authors did not include sub‐analysis data describing; time since orchidectomy, PSA levels specific to type of GAHT/orchidectomy, or GAHT doses. However, the antiandrogen medications used were the same as those used in the other three papers (cyproterone acetate and spironolactone), with the addition of 10 participants using medroxyprogesterone acetate. There were no PSA levels pre‐initiation of GAHT, and the mean PSA was taken at a single time point (no date available), where participants had received different regimens of GAHT over a mixed time span. The participants had a mean (standard error [SE]) serum PSA level of 0.1 (0.02) ng/mL, while the controls had a mean (SE) serum PSA level of 1.1 (0.1) ng/mL.

Obiezu et al. [23] was a prospective longitudinal study that featured a total of 56 trans women, divided into three groups according to type of GAHT, and with no inclusion of orchidectomy status. They measured mean/median PSA levels in trans women before initiation of GAHT, at 4 months and then 12 months after initiation. Only Group 3 (*n* = 31, mean [range] age 30 [18–34] years) reported a mean serum PSA level. This group received 100 μg oral oestradiol once daily and 100 mg cyproterone acetate oral antiandrogen once daily. The mean PSA levels at pre‐initiation, at 4 months, and 12 months were 0.327 ng/mL, 0.058 ng/mL, and 0.015 ng/mL, respectively. No SE values were given with these mean levels. For Group 1 (100 mg cyproterone acetate), the median PSA levels prior to commencing GAHT and at 4 months were 0.333 ng/mL and 0.035 ng/mL, respectively. For Group 2 (transdermal 17β‐oestradiol and 100 mg cyproterone acetate once daily), the median PSA levels prior to commencing GAHT and at 4 months were 0.272 ng/mL and 0.041 ng/mL, respectively.

Slagter et al. [24] was a prospective longitudinal study that featured a total of 35 trans women, with a mean (range) age of 30 (19–45) years divided into two groups. Group 1 contained 20 trans women who took 100 μg oral ethinyl oestradiol once daily and 100 mg cyproterone acetate once daily. Group 2 contained 15 trans women who took transdermal 17β‐oestradiol (equivalent to 100 μg daily) and 100 mg cyproterone acetate once daily. As for Obiezu et al. [23], the authors measured mean PSA in trans women before GAHT, at 4 months and 12 months after initiation. The mean (SE) serum PSA in all trans women before initiation of GAHT was 0.525 (0.044) ng/mL. At 4 months of GAHT, the mean (SE) serum PSA level for Group 1 was 0.032 (0.006) ng/mL and for Group 2 was 0.177 (0.072) ng/mL. At 12 months after GAHT initiation they reported no significant difference in mean PSA level, but the paper did not include raw levels.

Nik‐Ahd et al. [25] was a retrospective cohort study that measured 810 serum PSA levels in 210 trans women receiving oestradiol as GAHT (regardless of orchidectomy or other androgen‐blocking drugs). These data were found in the patient records of the Veterans Health Administration (VHA), the largest healthcare system in the United States. The study searched records dated between January 2000 and August 2023. The inclusion criteria were trans women aged >40 years without a diagnosis of prostate cancer who had a PSA test after receiving a minimum of 6 consecutive months of oestradiol therapy. The participants (*n* = 210) had a mean (SD, range) age of 60 (8, 40–79) years and had received oestradiol for a median (range) of 4.7 (0.5–29.9) years at the time of PSA testing. The median (IQR) PSA level was 0.02 (0–0.2) ng/mL. The highest PSA level was 2.21 ng/mL, the 95th percentile PSA level was 0.6 ng/mL, and 36% of participants had an undetectable PSA. Other variables such as age and race were also recorded. The authors reported PSA levels as a median, we contacted the authors requesting raw data to generate a mean average, but we were unsuccessful.

### Narrative Assessment of Heterogeneity

High heterogeneity across the four included studies makes meta‐analysis unfeasible. The four studies were varying in quality and risk of bias, with large variation in average age of participants, which is a dependant variable in the context of PSA levels and prostate cancer risk. Other dependent variables, such as length of GAHT use and type of GAHT regimen, also varied. It was not possible to compare different averages given both mean and median values were used. We assume the authors chose to use median where the PSA levels did not abide by normal distribution. The studies did not release demographic data for the participants, and we were unsuccessful in requesting these from the authors, therefore we could not perform sub‐group analyses.

### Comparability

All four studies measured serum PSA in TWNBPP who received GAHT, with the type and dose of GAHT similar across all the included studies.

Three studies used immunofluorometric assays for laboratory PSA testing, Nik‐Ahd et al. [25] did not specify this.

Obiezu et al. [23] and Slagter et al. [24] were comparable in design. They measured mean PSA level prior to commencing GAHT, and at 4 and 12 months after GAHT initiation. The mean PSA levels in both studies decreased from baseline after 4 months of GAHT, and Obiezu et al. [23] reported a further reduction at 12 months. These two studies also used similar sample sizes and had the same average age of participants.

Jin et al. [22] measured serum PSA levels at a single timepoint irrespective of the duration of GAHT treatment, with a median of 9 years use of GAHT and they did not give a date for the timepoint used. They used comparison to controls instead of comparison to pre‐GAHT PSA levels. The mean age of participants was between that of the other included studies.

The Nik‐Ahd et al. [25] differed in study design from the other three studies. The PSA results were collected across a 23‐year period for a large cohort. There was no control group or pre‐GAHT initiation PSA level; however, the paper used a comparison median (IQR) PSA level of 1.0 (0.6–1.9) ng/mL from ‘similar studies’ in age‐matched cis men. Finally, the reporting of a median PSA level may be a more reliable representation of average PSA than the mean used in other studies, if the distribution of values is skewed.

### Quality of Evidence

#### Age

Serum PSA levels increase with age and PSA reference ranges used for cis men are <2.5 ng/mL between the ages of 40 and 50 years and <4.5 ng/mL between 60 and 69 years [26]. The low average ages in Obiezu et al. [23] and Slagter et al. [24] were below the prostate cancer screening eligibility cut‐off for cis men, making them less relevant in prostate cancer screening. Using comparison to controls [22] reduces age as a confounding bias in the results, although the average age was still low and below the average age of prostate cancer diagnosis in TWNBPP. The average age in Nik Ahd et al. [25] was 60 years, which was closer to the average ages (61 and 64 years) in the two large studies describing prostate cancer in the TWNBPP population receiving GAHT, from which incidence and PSA at diagnosis data were taken [11, 12].

#### Prostate Pathology

These studies aimed to assess PSA levels in TWNBPP receiving GAHT who did not have prostate pathology (e.g., prostate enlargement, prostatitis, prostate cancer). They relied on participant self‐report of any prostate pathology, and did not include symptom assessment or imaging, and they did not perform prostate biopsy to definitively confirm absence of prostate cancer. This is a limitation when interpreting the PSA levels from the studies.

#### Sub‐Group Analysis

Only Jin et al. [22] and Nik‐Ahd et al. [25] reported demographic information about participants. They reported age, type and duration of GAHT, as well as orchidectomy status. This prevents sub‐group analyses to assess the role of confounding variables across the papers and investigations into causes of high heterogeneity.

#### Risk of Bias

Nik‐Ahd et al. [25] was evaluated as the most reliable and reproducible of the included studies, with the lowest confounding bias (Table 3). It was the only paper to state exact study time periods used, for the other studies we must assume they are contemporary to the years of publication. Study locations were only available for Jin et al. [22] and Nik‐Ahd et al. [25], the latter of which used data from multiple locations across the USA. Publication bias may have occurred given that trans health is a controversial topic, possibly deterring journals from publishing studies examining this area. This may have led to the low number of studies on the topic. Only Nik‐Ahd et al. [25] included their source of funding.

#### Overall Quality of Evidence

Using the GRADE tool (Table 4), the authors found the overall quality of evidence from the four studies to be very low.

### Utility of Evidence

All four papers supported the hypothesis that GAHT reduces PSA to levels <0.5 ng/mL, lower than that typically seen for cis men. Jin et al. [22] and Nik‐Ahd et al. [25] gave a PSA level that is applicable to a greater number of TWNBPP, and a larger sample size could be recruited if this method was replicated.

The evidence from the Nik‐Ahd et al. [25] paper had the greatest utility to achieve the goal of this review, which was to provide guidance for clinicians around PSA reference ranges for TWNBPP, and when to refer for further prostate investigations. The data were more representative of the study population due to a large sample size, wide location base, and older participants, with an average age of 60 years. This illustrates the effect of GAHT on serum PSA levels in those at risk of prostate cancer, as studies of prostate cancer in TWNBPP have reported the average age of diagnosis is in the seventh decade [11, 12, 20]. Nik‐Ahd et al. [25] adds the most value to evidence for PSA thresholds in TWNBPP receiving GAHT, as it accounted for 72% of the data in this review and it had the lowest risk of bias. Although the evidence does have limitations, which are the lack of a control group for comparison and the authors did not analyse PSA levels according to GAHT type. This is relevant because different GAHT regimens may confer differing degrees of testosterone suppression or blockade. It is possible that given the increased age of participants (≤79 years) and short median length of oestradiol therapy (4.7 years), there could be pre‐existing prostate cancer in some of these trans women, which cannot be confirmed in the absence of follow‐up data. These PSA levels cannot, therefore, be used as reference intervals.

Jin et al. [22] reported a mean serum PSA level in those who had received a median 9 years of GAHT, which has the benefit of demonstrating a more long‐term estimate of PSA levels in those receiving GAHT, especially with comparison to age and location‐matched controls, which reduces age as a confounding factor. Obiezu et al. [23] and Slagter et al. [24] have some utility in demonstrating a reduction in PSA level within 12 months of starting GAHT, but the high risk of bias and young participants make them less useful to guide clinicians around when to refer TWNBPP for further prostate investigations.

## Discussion

Currently, there are no clinical guidelines for clinicians around PSA level thresholds to trigger further prostate investigations in TWNBPP receiving GAHT, who are at risk of, or who are being investigated for the presence of, prostate cancer. There are two contexts in which a guideline would be clinically useful. First, a TWNBPP presenting with symptoms that may be suggestive of prostate cancer. Second, a TWNBPP who has risk factors, who may wish to discuss the role of PSA testing. Increasing age is the predominant prostate cancer risk factor. According to the literature, TWNBPP on GAHT are more at risk of the disease if they commenced GAHT after the age of 40 years [9, 11, 12, 27]. This is likely due to the possibility of pre‐existing prostate cancer when commencing GAHT and reduced time of testosterone suppression, assuming that testosterone suppression is protective against developing prostate cancer. Of course, TWNBPP who choose not to access GAHT, likely retain a similar risk profile to cis men. Other prostate cancer risk factors are: having a strong family history of the disease (especially if relatives were diagnosed below the age of 70 years), possessing a genetic variant in a DNA‐repair gene (carrying a pathogenic variant in *BRCA2* has up to an 8.6‐fold increased risk of prostate cancer), and Black ethnicity (doubles both prostate cancer incidence and mortality) [28, 29, 30, 31].

Evidence supports a lower incidence of prostate cancer in TWNBPP and we acknowledge that TWNBPP receiving GAHT, who possess risk factors, remain at lower risk of the disease than cis men. Therefore, we do not advocate for prostate cancer screening for all TWNBPP, but instead, individualised risk–benefit discussions. TWNBPP are still at risk of prostate cancer, and those who are symptomatic for the disease may require further investigations, including interpretation of a PSA test result. There are reports of more aggressive disease in the TWNBPP population, so an awareness is important. If a national screening programme was set up to include risk factors such as ethnicity, family history and genetic risk, guidance would be needed to advise on the management of this group of patients [32].

Review of the published literature up until July 2024 found only four papers reporting expected PSA levels in the TWNBPP population without prostate cancer and they are unsuitable to guide clinical recommendations. They supported there being a progressive reduction of PSA from initiation of GAHT until 12 months and suppression of PSA levels with long‐term GAHT use. They also demonstrated significantly lower PSA levels compared to age‐matched cis controls, even in older age brackets. Only Nik‐Ahd et al. [25] provided data in TWNBPP whose age put them at risk of developing prostate cancer, which in addition to largest sample size, and highest quality, makes this paper the most useful to guide clinical decision making. The issues with the literature are the small number of studies, many of which had low or very low certainty, issues with methodology, high risk of bias, and with large variation between studies. We can only assume that the participants had not received a diagnosis of prostate cancer; however, this was not confirmed on imaging or biopsy. The only prostate cancer risk factors included in the participant data was age. There were no ethnicity data included in the studies, which is relevant given those of certain ethnicities have greater prostate cancer risk.

This highlights the lack of robust evidence on both this topic and research more widely in early cancer detection in trans people. Authors have previously suggested 0.65–1 ng/mL as an upper limit for TWNBPP, although these limits are extrapolated from data in hypogonadal cis men [10, 33]. Hypogonadal cis men may share low serum testosterone levels, but sample sizes were small, and they did not receive exogenous oestrogen as is the case in TWNBPP on GAHT.

Prostate‐specific antigen reference intervals for TWNBPP on GAHT are lacking and the data from these four papers were of low quality and insufficient to guide clinical recommendations. Consequently, clinicians have little guidance around when to reassure TWNBPP regarding their prostate cancer risk after a PSA test or when to refer TWNBPP for further prostate investigations. This risks harm from overdiagnosis of low‐risk disease or harm from delayed diagnosis of high‐risk disease. Understanding clinically relevant PSA cut‐offs for investigation in TWNBPP receiving GAHT will be important to achieve a balance of risk–benefit in a population who is already disadvantaged in health outcomes [34].

Establishing PSA reference ranges for TWNBPP symptomatic of prostate cancer is important, as we work towards the incorporation of targeted screening methods for high‐risk individuals in primary care [34, 35, 36, 37, 38]. Prostate cancer screening using serum PSA is a controversial topic in cis men and is an unspoken topic in TWNBPP. There is no international consensus on prostate cancer screening. In advanced prostate cancer (high Gleason Scores), PSA sensitivity is 80–90% [39]. There are large data sets that report that PSA screening reduces mortality by ~20% [40, 41]. De Vos et al. [42], with a 21‐year follow up in 42 376 cis men, found that the rate ratio of prostate cancer specific mortality was 0.73 (95% CI 0.61–0.88) favouring screening. For metastatic prostate cancer, the rate ratio was 0.67 (95% CI 0.58–0.78) favouring screening [42]. However, screening with a PSA test alone cannot distinguish between clinically significant or indolent prostate cancer, which can result in unnecessary prostate biopsies, overtreatment (with potentially life changing side effects of incontinence and erectile dysfunction), and psychological burden [35]. Some argue these harms outweigh the benefit of mortality reduction.

Clinicians are less likely to discuss prostate cancer screening with TWNBPP individuals who have risk factors [4]. PSA testing in the trans community depends on shared decision‐making between TWNBPP and clinicians regarding the benefits of a PSA test. It is likely that many TWNBPP and clinicians are unaware of the prostate cancer risk in TWNBPP, and they may not know that the prostate is retained after GAS. TWNBPP with prostate cancer may present late due to fear of addressing their symptoms, which is contributed to by dysphoria and anxiety around discussing anatomy that is incongruent with their gender identity [9]. If GAHT is commenced after the age of 40 years, it is possible that there is small volume prostate cancer prior to starting GAHT, which is supported by papers revealing that TWNBPP who have received a diagnosis of prostate cancer received GAHT for shorter time periods and started GAHT later in life (compared to TWNBPP without a prostate cancer diagnosis) [9, 11, 12]. When prostate cancer is diagnosed in TWNBPP on GAHT, the cancer has arisen on a background of low testosterone, which in the case for cis men who present with castrate‐resistant disease, reduces prostate cancer treatment options and may reduce overall survival.

With minimal guidance around prostate cancer diagnosis in the gender diverse community, it is important to provide evidence where there currently is none; to enable clinicians to assess the prostate cancer risk in TWNBPP and request PSA and other investigations when appropriate.

## Conclusions

The literature regarding PSA levels in healthy TWNBPP on GAHT is sparse, of poor methodological quality, and low applicability. The four studies reviewed here support that GAHT reduces serum PSA levels below the individual's baseline PSA and below that expected for cis men of the same age. Only the Nik‐Ahd et al. [25] study provides evidence of an expected PSA threshold in TWNBPP receiving GAHT aged >40 years, without prostate pathology, whose age would fall within that appropriate for PSA testing. However, there is significant heterogeneity in these studies, and they do not inform clinical decision making. This review highlights an evidence gap in PSA reference ranges for guiding practice on when to initiate investigations for suspected prostate cancer for the gender diverse population.

## Disclosure of Interests

Professor Rosalind Eeles has the following conflicts of interest to declare: Honoraria from ‐ American Society of Clinical Oncology Genitourinary (ASCO GU), Janssen, University of Chicago, Dana Farber Cancer Institute USA as a speaker. Educational honorarium from Bayer and Ipsen, member of external expert committee to Astra Zeneca UK and Member of Active Surveillance Movember Committee. She is a member of the Scientific Advisory Board of Our Future Health. She undertakes private practice as a sole trader at The Royal Marsden NHS Foundation Trust and 90 Sloane Street SW1X 9PQ and 280 Kings Road SW3 4NX, London, UK. Dr Alison May Berner: Honoraria from non‐promotional education Gilead, Lilly, Astra Zenaca, Pfizer, Eisai, Astellas. Conference attendance subsidy from Gilead. Salary UK National Institute for Health Research (NIHR). Authors Rose Hall, Rue Ball, and Elizabeth Bancroft have no competition of interest to declare.

## Supporting information


Appendix S1.
