# Supplementary material for: Systematic review of PSA reference intervals in the gender diverse population with prostates
Source: BJU Int. 2025 Jun 22;136(4):568–77. doi: 10.1111/bju.16825 (PMC12415315; doi:10.1111/bju.16825)
Supplement: Supplementary file 1 — Appendix S1. [file BJU-136-568-s001.docx]

# Appendix S1. Search Strategy

# Database search

Databases searched: MEDLINE, Embase (via Ovid)
Search performed on: 16 July 2024

## Medline search 16/07/2024

("Transgender" or "Transsexual" or "Transsexualism" or "Gender dysphoria" or "Gender identity disorder" or "Gender diverse").af.
exp Transgender Persons/ or Transgender Persons.mp.
1 or 2
("Prostate specific antigen" or "PSA").af.
exp Prostate-Specific Antigen/ or Prostate-Specific Antigen.mp.
4 or 5
3 and 6
limit 7 to english

## Embase search 16/07/2024

("Transgender" or "Transsexual" or "Transsexualism" or "Gender dysphoria" or "Gender identity disorder" or "Gender diverse").af.
exp transgender/ or exp gender dysphoria/
1 or 2
("Prostate specific antigen" or "PSA").af.
exp prostate specific antigen/
4 or 5
3 and 6
limit 7 to English
